# Design Framework for Older Adult Health and Fitness Technologies: Proof-of-Concept Case Study

**DOI:** 10.2196/97010

**Published:** 2026-11-13

**Authors:** Christopher Tacca, Richard Gomer, Isobel Margaret Thompson, Arturo Vazquez Galvez, Chris Freeman, Martin Warner, Alexander Dawid Bincalar, Alexander Ng, m c schraefel

**Affiliations:** 1 School of Electronics and Computer Science, Faculty of Engineering and Physical Sciences, University of Southampton, Building 32, University Rd, Southampton, England, SO17 1BJ, United Kingdom, 44 23 8059 944; 2 School of Health Sciences, University of Southampton, Southampton, United Kingdom

**Keywords:** design frameworks, activity theory, elder physical fitness, fitness technologies, digital health, physical activity promotion, older adults, incidental interaction

## Abstract

**Background:**

Research on fitness technologies to support older adults has grown significantly. However, reviews of older adult fitness technologies found mixed results and their use in practice by older adults lags behind other age groups. Our previous work, a scoping review, showed a lack of alignment between proposed technologies and expressed needs and preferences of older adults. We present the Older Adult Fitness Design Framework aimed to address this gap and use a proof-of-concept case study of a “smart chair” as an example of the framework in use.

**Objective:**

This study aims to investigate how the framework can be used to help identify potential design gaps and inform the iterative development of older adult fitness technology interventions.

**Methods:**

The framework was developed by synthesizing known important older adult–specific factors from technology acceptance and qualitative literature and grounded in activity theory. The proof-of-concept case study consisted of four parts: (1) a 2-week pilot study, (2) 2 design workshops led by the use of the framework, (3) a 4-week follow-up study to assess the changes from the design workshops, and (4) 6 focus group and one-on-one interview sessions to explore more deeply design ideas stemming from the workshops. Usage rates and participant feedback were collected for the 2 pilot studies and thematic qualitative analyses of the focus groups were conducted. All studies were approved by the University of Southampton Ethics Committee.

**Results:**

The pilot study (male: n=1, female: n=2, average age 78, SD 10.44 years) suggested an overall positive reception to the “smart chair” intervention. However, lower scores on enjoyment and motivation suggested the need for further development. Design workshops identified changes related to space requirements, training materials, screen alternatives, and adding enjoyment and engagement. Results from the 4-week study (male: n=2, female: n=1, average age 70.3, SD 12.50 years) suggested a gap related to motivation and enjoyment in the screen-based design. Six focus group and interview sessions (total: 25 participants) were conducted in the local community. Feedback from these sessions suggested a preference toward an LED interface alternative, noting its simplicity, discreteness, and fun. Furthermore, discussions oriented around the older adult factors demonstrated how using the framework could potentially lead to nuanced feedback around motivation, enjoyment, privacy concerns, and stigma.

**Conclusions:**

We present a framework aimed at supplementing participatory design practice by synthesizing what is already known about older adults related to technology and health and allowing researchers to identify potential areas for improvement more easily through self-assessment. We encourage researchers to try it and give us their feedback, so that we can better support researchers in the space.

## Introduction

### Background

Research for the development of innovative fitness technologies to improve older adult fitness and support independence has grown significantly over the past decade [1,2]. The increase in interest in this area has spanned several different fields of research, including gerontology, medicine, public health, engineering, computing, and psychology, with the hope that development of health and fitness technologies can bring more accessible and preventative care to older adults, reduce the growing burden on the health and care sector, and ultimately, support older adults in living fulfilling and independent lives longer [3]. However, despite the growing interest, reviews of relevant older adult fitness technologies found mixed results in terms of evidence of effectiveness, adherence, and study quality [4-9]. Additionally, commercial use of health and fitness–related technologies by older adults has lagged other age groups [10].

Looking toward qualitative literature on older adult health and fitness technology preferences and older adult–specific technology acceptance literature can help provide a possible explanation for the lack of translation and overall low adherence. Qualitative research on older adult health technology preferences has shown mixed reactions toward wearables [11,12], a strong importance placed on privacy [11-13], and potential resistance related to identity such as stigma associated with needing to use these types of technologies [11,13-15]. In addition, related literature suggests that factors such as usability, value, social support, cost, and independence [16-18] all contribute to whether older adults are likely to adopt a new technology into their daily lives.

Our previous work, a scoping review and analysis of the space illustrates this point [19]. It shows potential issues related to privacy, overall compatibility with lifestyle and routines, ease of uptake, motivational elements, social engagement, and perceived dignity and independence. Concrete examples of the disconnect include overly complex technological systems, required 24/7 monitoring of users, rigid fitness programs that do not fit easily into established routines, and technologies that have large space requirements. The findings suggest that explicit considerations of these factors early in the design process could help to mitigate technology design issues and better position older adult fitness technologies for translation and ultimately better support older adults in practice [19].

This work is a follow-up to the scoping review to propose a design framework to address this gap and make it easier for people interested in developing fitness technologies for older adults to design technologies that more closely align with older adults’ expressed needs and preferences.

### Design Frameworks

Design frameworks have been used in research, particularly in human-computer interaction (HCI) literature, to help researchers by creating a specific design space to guide development of both research and implementation for any associated interventions. Existing design frameworks cover use of particular technologies including equitable and social wearables [20,21], ingestible sensors [22], reflective games [23], and mixed reality systems [24,25], as well as specific design practices and approaches including behavioral change technologies [26] and co-design [27].

Design frameworks are generally aimed at addressing the emergence of new technology, underserved or understudied population groups, or new theoretical design approaches. Researchers draw on established design theory, practical advice from in-the-field use, systematic reviews, and co-design with stakeholders to formulate design frameworks. Finally, researchers validate those frameworks through comparative studies to better understand if the framework is providing the desired effect in the field. Using a design framework for just-in-time diet and physical activity interventions [28] as an example, the framework was developed for body-worn sensors and through feedback from a systematic review. Another example [29], aimed to bring attention to technology design for people with attention deficit hyperactivity disorder (ADHD) and was developed through investigating existing technology approaches in the field and cross-analyzing them across established ADHD challenges. A third example, for computer-supported collaborative learning (CSCL) systems [30], draws on activity theory to orient the framework.

Design frameworks such as these have been used in the literature to guide both future research practice and design decisions for new technology development. Using Sonne et al [29] as an example, it has been cited as forming the basis for design guidelines of tabletop activities for children with ADHD [31], for specific design mechanism suggestions for online peer support for adults with ADHD [31], and as motivation for further research into the preferences and design considerations for people with ADHD [32]. Using Zurita and Nussbaum [30] as another example, it has been cited regularly as an evaluation tool for analyses of the designs of mobile-based learning systems [33-35], as a model for the use of activity theory for technology development in the online instruction space [36-38], and as an evaluation tool for specific computer-based teaching tools [39].

Following a similar approach, we propose a new framework, the Older Adult Fitness Technology Design Framework, to support researchers in creating older adult fitness technologies. The framework draws on existing literature on older adult technology needs and preferences including older adult technology acceptance factors [11,14,16,17], barriers and enablers to older adult fitness [40,41], and qualitative feedback from older adult fitness technology studies [12,13], orients itself through an activity theory lens (described below), and is explored through an initial case study. We propose that this framework for older adult health and fitness technologies could be beneficial for helping researchers better support older adults, a growing and currently under-supported group.

### Activity Theory

Activity theory offers a way to discuss how to connect the design and assessment of older adult health technologies with the needs and preferences of their users. The theory [42] introduces the concept of an “Activity,” a purposeful or transformative action conducted between actors (subjects) and the world (objects). Activities have two key characteristics: (1) the subjects of activities have needs and (2) activities and their subjects mutually determine one another. Take weightlifting, for example, one might suggest that a person (subject) can lift a heavy weight (object) only if that person is strong enough; however, activity theory points out that the causal relationship could be considered in reverse: the process of weightlifting (activity) determines the strength of the person (subject). Activity theory has extended beyond both geographical and disciplinary borders and has emerged as a central theory in the field of HCI. Researchers such as Bødker, Kaptelinin, and Nardi [42-44] have worked to adapt and develop the theory into a fundamental conceptual framework for HCI principles. Activity theory has been used as an object of analysis for further development of the theory [45-48], the development of new analytical tools such as design frameworks or evaluation tools [45,49,50], and as a tool for empirical evaluation in specific contexts such as health care and education [45,51,52]. For the purposes of this manuscript, we will focus on its use as a framework for design.

The key contribution of activity theory toward technology design was to extend the field’s scope of analysis and subject matter. With activity theory, the use of technology is embedded in meaningful context, not limited to information processing, and operates at several levels that have to be integrated. This suggests that interaction design must extend beyond considering only the simplest forms of system functionality and usability and also consider the interests, emotions, hopes, passions, fears, and frustrations of the potential users [44]. In an effort to synthesize the design principles of activity theory in HCI, Kaptelinin, Nardi, and Macaulay developed the activity checklist [24], which provides guidelines for design with activity theory.

Activity theory also offers a lens for examining technology interactions through “affordances,” or what the environment offers the subject [43]. Looking at affordances can help predict how someone new might interact with a proposed technology and can help illuminate ways in which the researcher can make their own technologies more user-friendly and have the intended effect. Features such as a removable back cover of a phone, a headphone jack, or a scroll bar all can be described as providing a different type of affordance [43].

### Research Question

This manuscript reports the development of the framework, a proof-of-concept case study using the Elder Smart Chair (a smart chair for encouraging sit-to-stands), and the major takeaways from the case study as they relate to the framework.

The research question investigated in this study is as follows: How can the Older Adult Fitness Technology Design Framework be used to help identify potential design gaps and inform the iterative development of older adult fitness technology interventions?

## Methods

### Overview

In this section, the methodology for the framework development and proof-of-concept case study is discussed.

### Identifying Important Design Considerations for Older Adult Fitness Technologies Success

Based on the previous scoping review that synthesized older adult–specific technology acceptance [19], older adult barriers and enablers to physical activity, and qualitative feedback from older adult fitness technology literature, 6 design considerations were identified and included in the framework. The six design considerations are as follows:

1. Compatibility with lifestyle: How well a fitness technology intervention fits into the potential user’s overall lifestyle including daily routines, habits, preferences, and values. The ability for a technology to seamlessly fit into older adults’ lives has been found to be an important factor for older adults [16]. Poor accessibility was found to be a barrier to older adults’ physical activity and exercise [40,41].
2. Similarity with past experience: How similar technologies are to the older adults’ previous experiences and how well training, education, and personalization are incorporated into the intervention. In both older adult–specific [17] and acceptance literature for the general population [53], past experience has been identified as an important factor. Specifically, literature suggests that technologies that are built on prior knowledge are more easily adopted [14,16] and older adults showed a preference toward “augmenting current technologies” [11].
3. Dignity and independence: Whether the health intervention, user interface and verbiage, and technology itself promotes a feeling of self-sufficiency and independence in its users or promotes stigmatization and a perceived dependency on technology. Identity is a major factor in acceptance of health-related technologies [13]. Technologies should avoid including language or approaches that stigmatize users and avoid making assumptions about potential users’ capabilities [14]. Older adults also tend to resist assistive technologies that make them feel old, helpless, dependent, or incompetent [13]. Autonomy and independence were also found to be important acceptance factors [13].
4. Privacy: How well the user’s privacy concerns related to personal information and data are respected. Older adults identified privacy as a major concern in accepting health-related technologies [11]. Related to health wearable technologies, there was widespread concern about data privacy and storage and the continuous monitoring associated with those technologies [12]. In addition, some older adults reported a preference for only tracking data relevant to their health condition [12]. Finally, older adults expressed concerns about technologies such as wireless sensor networks, bed occupancy sensors, and fall detection monitors for making them feel like they were always being watched or spied on [13].
5. Social engagement: The ability for the health intervention and technology to encourage or support social connection. Acceptance literature found older adults more willing to adopt a technology if family, friends, or community members also used the technology [16,17]. The same has also been found in literature on barriers and enablers to physical activity and exercise [40,41]. Social engagement is also positively linked to usability and learning as older adults tended to prefer turning to family or peers for help with technologies [11].
6. Emotion: The ability for the intervention and technology to be motivating, fun, or emotionally stimulating. Emotion has been found to be an important factor for both technology acceptance [17,53] and enabling physical activity [40]. Factors such as motivation [40], confidence [40], novelty [40], gamification [41], sense of achievement [41], and rewards [41] all positively help to enable physical activity and exercise.

More information on how these design considerations were synthesized can be found in Tacca et al [19].

### Adapting Activity Theory to Older Adult Fitness Technologies

Using the concepts of activities and affordances from activity theory, more complex technological systems can be summarized into the key components that the user interacts with and the mechanism by which it aims to prompt that interaction [45,54]. The summarization process for the framework based on activity theory is described here.

Five themes are used to categorize interventions:

1. Critical technology: the technology that users directly interact with,
2. Interaction type: the proposed method of interaction that a user would experience in the intervention (ie, was it an exergame or was it personalized advice by a professional?),
3. Behavioral lever: the intended mechanism for behavioral change,
4. Goal: the immediate behavioral change goal (eg, train balance), and
5. Overall objective: the overall goal that the intervention is trying to accomplish (eg, reduce falls).

For the behavioral lever theme, three possible categories were considered: (1) motivate, (2) inform, and (3) make more accessible. The distinction between goal and overall objective provides a useful way to recognize commonalities between interventions across disciplines. For example, interventions aimed at rehabilitation, fall risk reduction, and sarcopenia treatment may all ask patients to do exercises for building strength. Thus, the patient experience would be quite similar even with disparate overall health goals, described in this manuscript as the overall objective.

Using Nawaz et al [55] as an example, the summary could be: using an MS Kinect (1) to facilitate an exergame (2) to motivate (3) users to build strength and train their balance (4) in order to reduce fall risk (5). Another example summarizing an intervention by de Batlle et al [56] could be the following: Using wearable sensors and a website (1) to facilitate personalized health advice (2) to inform (3) users on ways to increase their physical activity (4) to help chronic illnesses (5).

### Proof-of-Concept Case Study for Framework Overview

To explore the use of the Older Adult Fitness Technology Design Framework, a case study was conducted on our existing older adult fitness technology system: the Elder Athlete Smart Chair. The case study consisted of four steps:

1. An initial 2-week at-home pilot study,
2. An evaluation of the results of the initial study and iteration of the design using the framework,
3. A follow-up 4-week study using the new technology system, and
4. Focus group events to investigate future design ideas based on the framework discussion.

### Ethical Considerations

The 3 studies (pilot study, follow-up study, and focus groups) were reviewed and approved by the University of Southampton Ethics Committee (ERGO #’s: 95,649 .A1, 99,702 .A3, and 108805). Following participation in the study, all data collected were anonymized and each participant was given a unique identifying number. Each participant was given a gift card as compensation for their participation. Consent forms used in the case studies can be viewed in Multimedia Appendices 1–3 .

### Elder Athlete Smart Chair

The technology chosen for the case study is from an existing project as part of the WellthLab at the University of Southampton, the Elder Athlete Project [57]. The goal of the project is to develop technologies that support older adults to build strength and maintain their independence by doing things that they are already doing in a way that helps build and maintain strength and balance. The specific technology investigated in this case study is one component of that system, the Elder Athlete Smart Chair. The smart chair is a set of sensors that can be placed on any living room chair in an older adult’s home and an accompanying monitor that detects how often the older adult stands up from the chair throughout the day and gives visual feedback to encourage them to stand more often. The basic approach is to encourage older adults to “do it twice,” in this case, encourage older adults to, any time they stand up, sit back down and stand up again, in other words, complete a sit-to-stand “double.” Features of the system include a “singles” and “doubles” daily counter, daily and weekly goals, daily and weekly summary pages, recordings of how long it takes older adults to stand and whether older adults use their hands, and space for older adults to record their current state on a 1 to 5 bad-to-good Likert scale. A diagram and image of the Elder Athlete system is shown in Figure 1. The initial design of the smart chair was done with knowledge of older adult–specific technology preferences, but the Older Adult Fitness Technology Design Framework was not used until stage 2 of the case study.

**Figure 1.**
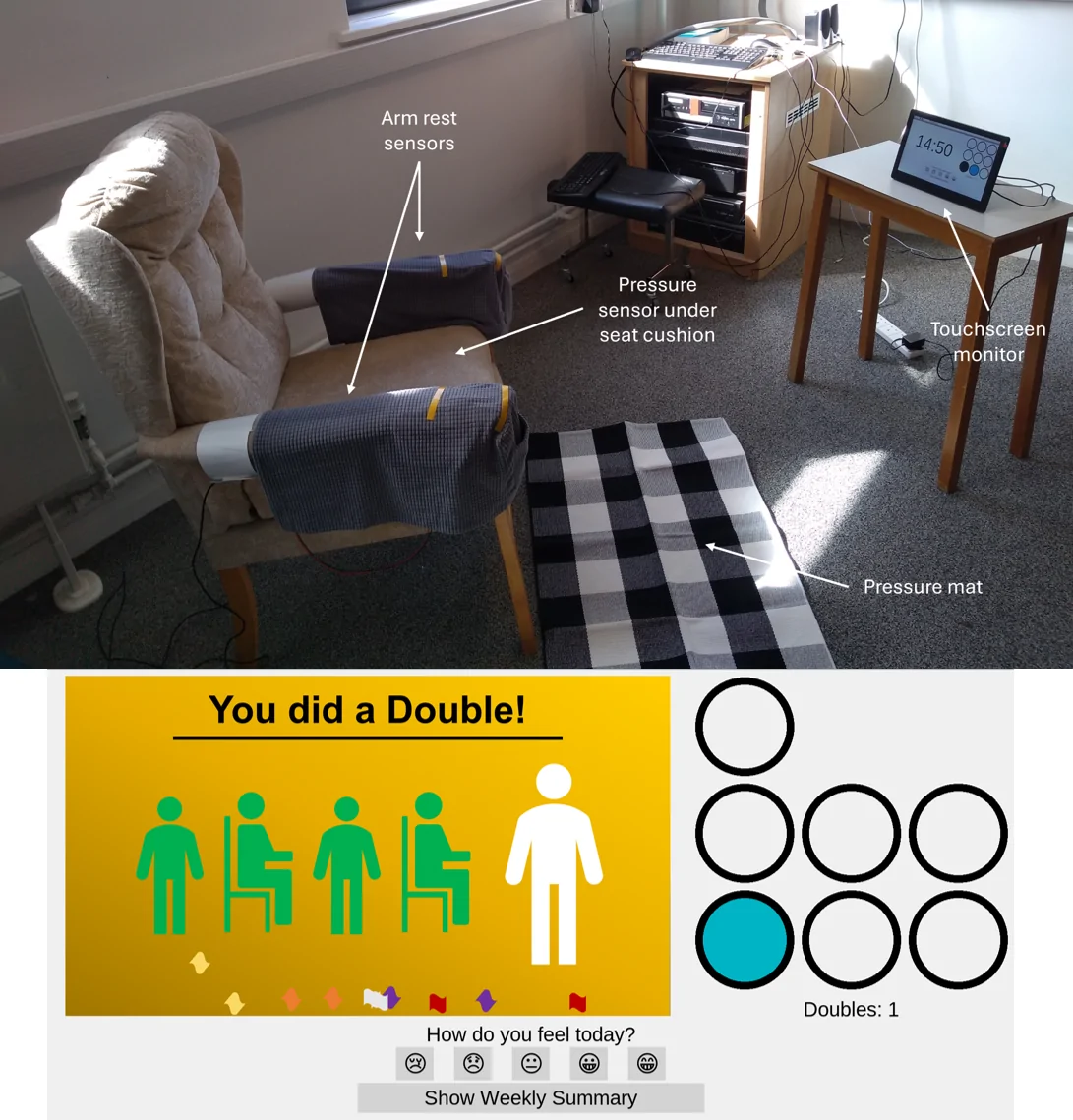
Image of the initial elder athlete system including the smart chair sensors to track when a user is sitting or standing, pressure mat to monitor users’ balance, and a touchscreen to provide encouragement and summaries of daily and weekly activity.

### Pilot Study

Prior to conducting a pilot study, the research team conducted preliminary in-laboratory studies to test the feasibility, functionality, and usability of the system. The pilot study was the first at-home trial to test the system’s robustness. Participants had the “smart chair” installed in their home and were asked to use it as they pleased for 2 weeks. The criteria for inclusion were as follows: English-speaking individuals over the age of 65 years with a score of 6 or below on the Clinical Frailty Scale [58] and physically able to complete repeated standing and sitting exercises. Study metrics in this trial included usage metrics: how many sit-stand doubles completed per day and how many daily goals met, before and after strength metrics: Physical Activity Scale for Elderly (PASE) [59], EQ-5D-5L Health State [60], 5 Times Sit to Stand [61], Timed Up and Go (TUG) [62], Hip Flexor strength, Knee Flexor strength, and Knee Extensor strength; and Feedback Surveys: Usability, Usefulness, and older adult fitness technology-related qualitative feedback questions. The survey questions were developed specifically for this study to explore older adult–specific factors and intended to be descriptive in nature. Participants rated their experience from 0 to 10 in the following way and scores were averaged for each question in their respective category:

1. Compatibility
  - How well did the intervention align with your personal fitness goals?
  - How easy was it to incorporate into your daily routine?
  - How much effort did you need to put in to stay consistent?
  - Overall, how intrusive was it to your daily life?
  - To what extent did it allow you to maintain your usual activities?
2. Similarity
  - Do you believe the intervention provided you with the necessary tools and knowledge to continue building your strength?
  - To what extent did the intervention resemble any previous programs or methods you have used for strength training or physical activity?
  - How easy was it to navigate any technology or platforms used?
3. Dignity and Independence
  - How well did the intervention support your independence in managing your strength-building or physical activity efforts?
  - To what extent did the intervention respect your personal preferences?
  - How trustworthy did you find the intervention overall?
4. Privacy
  - Overall, how well did the intervention respect your privacy?
5. Social Engagement
  - To what extent did the intervention provide you with social support that positively impacted your experience?
6. Emotion
  - Did you feel more motivated to engage in physical activities after the intervention?
  - How emotionally stimulating did you find it?
  - To what extent did you enjoy participating in it?
  - To what extent did it feel physically demanding to you?
  - Did it feel tiring to complete?

Other questions were related to general perceived effectiveness, technical performance, and open-ended feedback. While strength metrics were collected during the initial pilot study, they are not reported in this manuscript as the focus for this paper is the participant usage and feedback.

### Use of Design Framework

Following the pilot study, the project team met for 2 design workshops focused on using the Older Adult Fitness Technology Design Framework based on the feedback from study participants. During the workshops, researchers on the team first individually worked through the framework and then met together to have a group discussion of the major takeaways and what design changes could be made to address the feedback from the pilot study. The group was charged with proposing specific actionable design and protocol changes that would be implemented in the system for the follow-up study.

Actionable changes were categorized and prioritized into two groups:

1. Immediately actionable and minimal changes that could be implemented and investigated further in a similarly structured follow-up study.
2. Larger changes that more drastically altered the user interaction and needed to be explored further with more open and qualitative feedback in the form of focus groups.

### Follow-Up Study

The follow-up study was structured in the same way as the pilot study; however, participants were asked to keep the system in their homes for 4 weeks instead of 2, because the focus of this study was to measure engagement over a longer period. The same criteria for inclusion and study metrics were used including usage metrics, strength metrics, and qualitative feedback. While strength metrics were collected in the follow-up study, they are not reported in this manuscript as the focus for this paper is on the participant usage and feedback.

### Focus Groups and One-on-One Interview Sessions

To investigate the design changes suggested from the framework discussion that necessitated more qualitative feedback, the design team held 3 focus group events and 3 one-on-one interview sessions with older adults in the community. The largest focus group event was conducted at a local community center, and all other focus group and interview sessions were conducted in the laboratory. Focus group events consisted of a demonstration of the various prototypes and design mockups with the invitation for participants to try out the prototypes, followed by an open discussion to receive both participant feedback and design ideas. The discussion questions were developed based on the Older Adult Fitness Technology Design Framework and were aimed at supporting an open dialogue between the older adults present. One-on-one interview sessions were conducted in a similar manner. First, participants demoed the prototypes and mockups with specific questions for each one. Then, participants were asked overall discussion questions to allow for open feedback. The mix of focus group and individual sessions was done to accommodate as many older adults from the community as possible and to see whether similar feedback was found from the differing contexts. The specific discussion questions can be viewed in the Multimedia Appendices 1–3.

The prototypes and mockups were all different ideas for ways to encourage older adults to do more sit-to-stand doubles throughout the day. The specific items investigated in the focus groups, shown in Figure 2, are the following:

1. Screen-based interaction (same as pilot studies),
2. Light-based systems that follow a sequence for sit-stand doubles, and
3. Various mockups for prompting discussion on shape, size, and design of potential designs

Focus group sessions were recorded, transcribed, and analyzed using thematic analysis.

**Figure 2.**
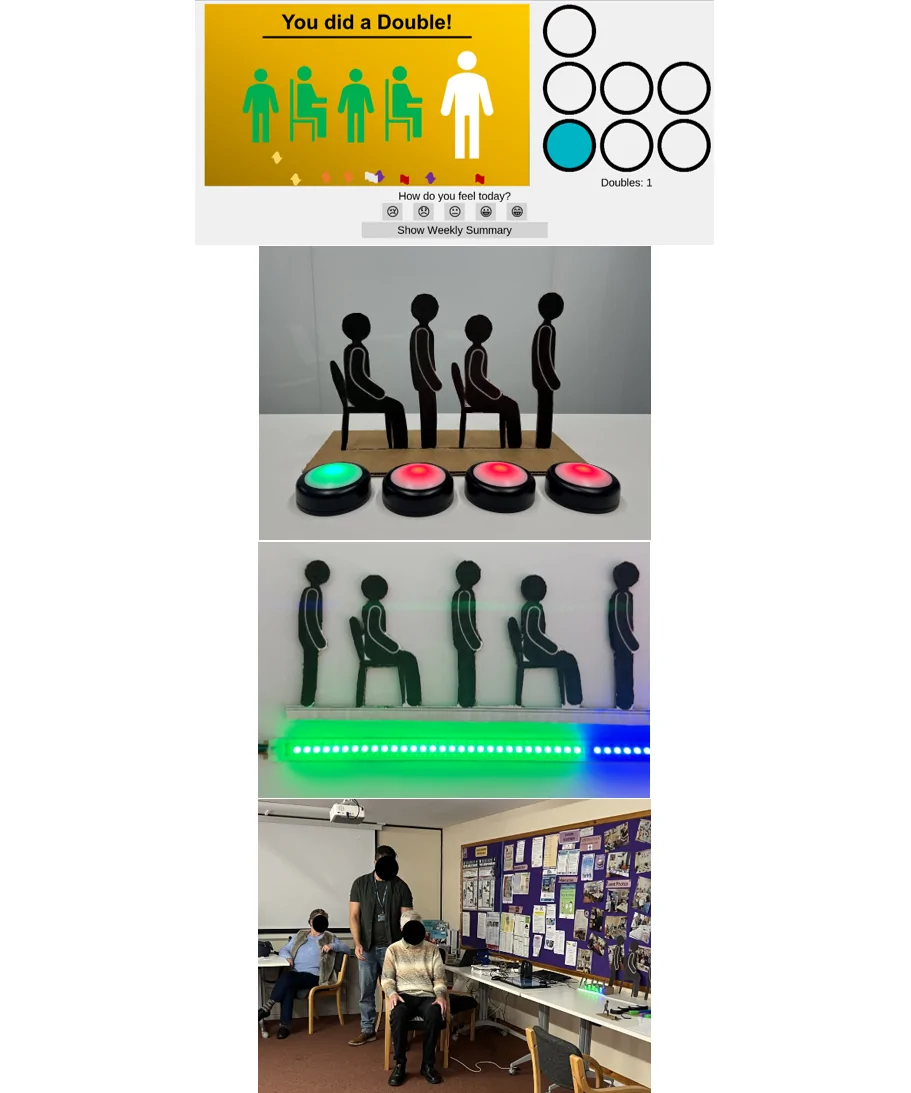
Images of the prototypes and mockups presented during the focus group events. From top to bottom: (1) screen-based intervention used in the 4-week usability study, (2) mockup of potential light sequence-based design with stick figures that were used as placeholders for both static and dynamic figurines and sized-based questions, (3) prototype for LED-based sequence design that prompts users to do a double sit-to-stand in which the green are the stages that are already completed and the blue are the unfinished stages, and (4) the LED-based sequence design in use at focus group session at a local community center showing the current state of the user in green and prompting the users’ next action in blue.

### Qualitative Analysis of the Focus Groups and Interviews

Audio recordings from the focus groups and interviews were transcribed manually and the qualitative analysis was conducted by 2 separate coders. Each individual used the same standard thematic analysis protocol for qualitative research [63] to identify the keywords, codes, and overall themes from each session. Themes were generated from the codes using an inductive approach and intended to be descriptive. Due to the nature of the discussion questions, themes were mapped to the older adult–specific factors in the framework when discussion questions or participant responses specifically referenced a given factor. Following the individual qualitative analysis, the 2 coders met to resolve any discrepancies in the analysis via consensus.

## Results

### Overview

Results reported in this study include the Older Adult Fitness Technology Design Framework itself, results from the initial 2-week study, summarization of the outcomes from the design workshops using the framework, results from the 4-week follow-up study, and results from the focus group events.

### Older Adult Fitness Technology Design Framework

As shown in the Multimedia Appendix 4, the framework consists of three parts: part 1: summarization of the health intervention through an activity theory lens, part 2: assessment of the design across 7 design considerations (the 6 considerations described in the introduction and health or behavioral outcomes), and part 3: plan of evaluation of the intervention.

In part 1, researchers are given a short explanation of the 5 categories used to describe the health intervention, critical technology, interaction type, behavior lever, goal, and overall objective with a space to fill in their own words what that would be for their intervention. After filling in each of those boxes, researchers are asked to summarize the intervention using the following sentence, “Using (critical technology) to facilitate a/an (interaction type) to (behavioral lever) older adults to (goal) in order to (overall objective).” As an example, a researcher developing an mHealth app to encourage older adults to walk more may fill in the sentence like this, “Using a smartphone app to facilitate a self-tracking health intervention to motivate older adult adults to increase their physical activity in order to maintain their independence longer.” Summarizing the interventions in this way may help researchers separate the technologies that are necessary for the systems to function from the ones that older adults will need to interact with, ground their decision-making for the types of interactions they are intending for older adults to have in the context of older adults’ lives, and isolate the why for the intervention in the first place. When assessing interventions in part 2, having this explicitly stated could catch inconsistencies between the intention and its use in practice.

In part 2, researchers assess their interventions across the 6 factors described in the Methods section with the addition of an outcomes factor, which is included to prompt researchers to consider the available clinical evidence of the intervention’s efficacy, described in more detail below. The specific tasks asked of researchers for each factor and their rationale will be explained here.

The first factor, outcomes, asks researchers to identify the established health outcomes for similar health interventions in the field with the same identified goals, then identify whether their intervention is aligned with those identified outcomes, and finally in the case they are not, change the intervention to match.

Following the same pattern, the second factor, similarity with past experience, first asks researchers to write the technologies that older adults will directly interact with, then assess whether older adults are familiar with those technologies and if they can point to evidence that suggests they are, and finally make the requisite changes if they are not. For this factor, that could mean switching the technologies used, designing the interaction of the system to be more similar to something older adults are familiar with, or developing training materials for how to interact with the unfamiliar parts of the system.

The compatibility with lifestyle factor asks researchers to consider how well the health intervention fits into the daily life of older adults in practice; this includes considering space requirements, disruptions to routines, flexibility of use, and equipment requirements. Researchers are asked to consider ways to make their interventions as minimally disruptive as possible.

Dignity and independence asks researchers to consider how much agency and control older adults have over how they will interact with the health intervention.

Privacy concerns ask researchers to think about the data they are collecting and try to minimize their data use to only what is necessary for the intervention to be effective.

Social engagement encourages researchers to identify ways to involve the community in the intervention, keeping social aspects entirely optional.

Finally, emotion asks researchers to consider how they can make their interventions more emotionally stimulating, motivating, and fun. For each factor, when researchers are unsure whether they have adequately addressed it with their current design, they are asked to make a note of it for part 3.

In part 3, researchers are asked to make a research plan for their health intervention. They are asked to consider the intended purpose of the study, intended users, duration of use for the system in practice, if co-design could support the research, and what the exit strategy is for the intervention. In addition, researchers are asked to take notes of assumptions and unknowns in part 2 and include them in their research studies either through a health metric or by asking them directly.

### Pilot Study Results

#### Overview

Three participants were included in the initial pilot study with 1 male and 2 female participants with an average age of 78 (SD 10.44 years, P1: sex: male, age: 73 years, initial EQ-5D-5L: 0.720; P2: sex: female, age: 90 years, initial EQ-5D-5L: 0.808; P3: sex: female, age: 71 years, initial EQ-5D-5L: 0.922).

#### Usage Results

Participant #1 (P1), male aged 73, participated in the intervention for a total of 14 days, and both participant #2 (P2), female aged 90, and participant #3 (P3), female aged 71 years, participated in the intervention for a total of 18 days. All 3 participants had an initial sit-to-stand goal of 20 sit-to-stands and 9 double sit-to-stands in week 1 and 32 sit-to-stands and 15 double sit-to-stands in week 2. For the single sit-to-stand goal, P1 met it on all 14 days, P2 on 8 of 18 days, and P3 on 17 of 18 days. For the double sit-to-stand goal, P1 met it on 10 of 14 days, P2 on 17 of 18 days, and P3 on 17 of 18 days. A detailed summary of their usage results is included in Table 1 and Figure 3.

**Table 1.** Summary of usage, average stands/day each week for each participant, results from 2-week pilot study.

Participants	Average stands/day, week 1, mean (SD)		Average stands/day, week 2, mean (SD)		Daily goal, week 1 (% met)		Daily goal, week 2 (% met)	
	Single	Double^a^	Single	Double^a^	Single	Double^a^	Single	Double^a^
1	35 (14.8)	11 (7.2)	51 (6.4)	18 (3.1)	20 (100)	9 (57.1)	32 (100)	15 (85.7)
2	21 (0.9)	9 (0)	30 (4.4)	14 (2.3)	20 (87.5)	9 (100)	32 (10)	15 (90)
3	25 (3.3)	10 (1.3)	38 (5.0)	16 (1.1)	20 (87.5)	9 (87.5)	32 (100)	15 (100)

a Double stands refer to two consecutive sit-to-stands (<15 seconds between stands).

**Figure 3.**
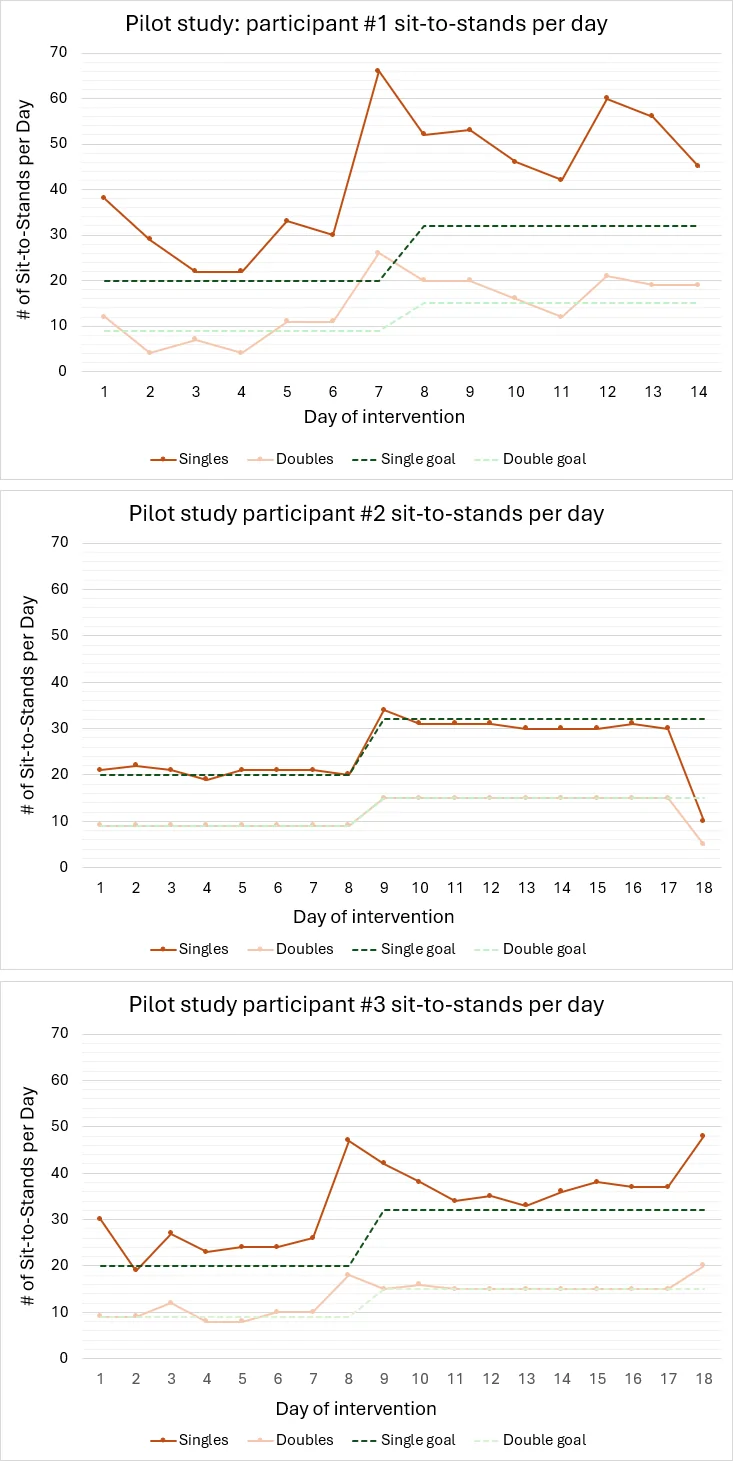
Sit-to-stands per day for the 3 participants in the pilot study, top to bottom: participant #1, participant #2, and participant #3. Shows single sit-to-stands, double sit-to-stands, and their respective daily goals.

#### Usability Survey Results

All 3 participants rated privacy relatively positively (P1: 8, P2: 10, and P3: 10). While P1 and P3 rated compatibility relatively positively (P1: 6 and P3: 7.40) and P2 and P3 rated dignity and independence (P2: 6.33 and P3: 7.67), technical performance (P2: 7 and P3: 7), and effectiveness (P2: 6 and P3: 6) relatively positively. Only P1 rated any factor below a neutral score of 5 out of 10: social engagement (0), emotion (3.40), effectiveness (4), and technical performance (3), respectively. A detailed summary of his usability survey feedback results is included in Table 2.

**Table 2.** Summary of usability survey results from pilot study. Survey prompts were rated on a scale from 1 to 10 and categorized into the respective older adult preference factors.

Participants	Compatibility^b^	Similarity^b^	D and I^a,b^	Privacy	Social	Emotion^b^	Effectiveness^b^	Technical performance
1	6	6	5.67	8	0	3.40	4	3
2	6.20	5	6.33	10	5	5	6	7
3	7.40	4.33	7.67	10	2	3	6	7

a D and I refers to the dignity and independence factor.

b Scores averaged from multiple prompts: Compatibility (5), Similarity (3), D and I (3), Emotion (5), and Effectiveness (2).

#### Open Feedback

In their open feedback, P1 suggested that we make the system movable because currently there are days when he is not or does not want to be using the chair where it’s set up, P2 felt that the setup was a bit intrusive in her home, but that there were no challenges in completing the intervention, and P3 suggested that an audible reminder would be useful, and that a system that required fewer wires would be less intrusive in the home when she had guests over.

### Design Workshop Using Framework Results

#### Overview

Two design sessions were conducted following the initial pilot study to discuss the results and plan improvements to the Elder Smart Chair using the framework. Here is a summary of the results from the 2 design sessions.

#### Summary of Interaction

The intervention was summarized with the following sentence: “Using a smart chair, touch screen, and pressure mat (critical technologies) to make an incidental encouragement intervention (interaction type) to motivate and suggest (behavioral lever) older adults to increase their number of sit-stands throughout the day (goal) in order to build strength and maintain their independence (overall objective).”

#### Consideration of Older Adult Specific Factors

The results from part 2 are summarized in Table 3. Overall takeaways from this portion of the design sessions included suggesting the removal of the pressure mats that were taking up too much space in the users’ living rooms, adding a toggle-off feature to the daily goal in order to avoid pressuring users to do their exercises immediately, considering adding an optional leaderboard for friends and family, and finally considering removing the screen entirely and replacing it with a physical figurine. In addition, further investigation was needed to determine if the usage continues in the long term and if use of the intervention leads to growth in lower body strength.

**Table 3.** Summarization of the results from part 2 of using the framework during the design sessions. Included in this table are the design groups’ decisions on the evidence from the initial pilot study, suggested changes, and still present assumptions and unknowns.

Older adult specific factor	Evidence from initial trial	Suggested changes	Assumptions and unknowns
Outcomes	Participants consistently met their daily sit-to-stand goals, increased their sit-to-stand speed; however, there were inconclusive results related to health state and strength measurements. Literature evidence suggests that with longer and more consistent use, the increased number of sit-to-stands throughout the day would increase lower body strength [64].	No suggested changes	Further investigation is needed for longer-term effects on strength and independence
Compatibility	One participant did not have a chair suitable for all system functionalities (no arms, thus no ability to install arm rest sensors). Overall score on compatibility-related survey questions was 6.53; participants were free to use the system as they pleased, the pressure mat took up too much space, and all 3 participants tended to do all their sit-to-stands in the morning	Make pressure mat completely optional Update the demo and training materials Update monitor UI^a^ to encourage using the system naturally throughout the day (include an option to hide the daily goal) Explore alternatives to the screen entirely such as a physical figurine Consider encouraging users to rest after 3 doubles in a row	Would older adults find the system disruptive to routines in the long term? Is continuously displaying the goal leading to older adults feeling pressured to complete the sit-to-stands immediately? What would the interaction look like without a screen? Would the effects of the intervention be less salient over time?
Similarity	Smart chair system: interaction familiar as older adults need only to sit and stand as they normally would; however, in some cases, older adults needed to sit back in the chair for the results to be counted. Touchscreen: no reported issues using the touchscreen, but full features not often used such as the weekly summary	Explore alternatives to the touchscreen including a physical figurine Fix sensor setup to allow for different sized participants with different chairs to sit as they normally would	Can the touchscreen UI be improved for better usability?
Dignity and Independence	Older adults rated the system highly in survey questions related to dignity (6.56), older adults had mixed ratings on independence mostly related to if they believed the system would help them with their long-term independence	Make customizable display, allow older adults to increase their goal at any point (currently only available at the end of the week)	Will any of the feedback change over longer-term use if older adults have noticeable progress in strength
Privacy	Older adults scored the system highly related to their overall feelings of privacy using the system	Add privacy information to the manual such as who sees the data and what data they see	Will any of the suggested changes during this process affect privacy concerns?
Social Engagement	Currently, no social engagement feature is built into the system; however, participants expressed interest in using the system socially (with family and friends)	Optional: Add an optional social feature such as a friend’s leaderboard	Do they have Wi-Fi? Do they use a computer or smartphone? What type of social feature would they be interested in? Would adding a social feature compromise some older adults’ feelings of privacy?
Emotion	Participants rated the system low in terms of fun and enjoyment (3.80)	Consider other aspects such as sound, gamification, social engagement, etc to make the experience more enjoyable Recruit participants for whom sit-to-stands are more difficult to see if the results remain	Is the reason for the results that the intervention was too easy? Would a physical object versus a screen be more enjoyable? How can we build in early wins and signs of success?

a UI: user interface.

#### Assessment Plan

Following the design sessions, a 4-week follow-up study was planned to see if there were any differences in the interaction between participants over a period longer than 2 weeks.

### Follow-Up Study Results

#### Overview

Four participants were initially recruited for the 4-week follow-up study. One participant dropped out of the study after 2 weeks, so the usage rates and usability survey results were not collected. Therefore, 3 participants, 2 males and one female, with an average age of 70.33 (SD 12.50) years were included in the study (P1: sex: male, age: 56 years, initial EQ-5D-5L: 0.631; P2: sex: female, age: 76 years, initial EQ-5D-5L: 0.879; P3: sex: male, age: 79 years, initial EQ-5D-5L: 0.704). Despite being younger than the intended age group for the study and intervention, an exception was made for participant #1 (aged: 56 years) to be included in the study due to his lower mobility and desire to improve his strength and maintain his independence, the main objective of the Elder Smart Chair intervention.

#### Usage Results

Participant #1 (P1), male aged 56 years, participated in the intervention for a total of 27 days, and both participant #2 (P2), female aged 76 years, and participant #3, male aged 79 years, participated in the intervention for a total of 29 days. P1 had a daily goal of 32 single sit-to-stands and 9 double sit-to-stands, while P2 and P3 had a daily goal of 20 single sit-to-stands and 9 double sit-to-stands for the entirety of the intervention. For the single sit-to-stand goal, P1 met it on 8 of 27 days, P2 on 1 of 29 days, and P3 on 0 of 29 days. For the double sit-to-stand goal, P1 met it on 22 of 27 days, P2 on 0 of 29 days, and P3 on 0 of 29 days. A detailed summary of their usage results is included in Table 4 and Figure 4.

**Table 4.** Usage results, average stands/day each week for each participant, from the 4-week follow-up study.

Participants	Average stands/day, week 1 (SD)		Average stands/day, week 2 (SD)		Average stands/day, week 3 (SD)		Average stands/day, week 4 (SD)	
	Single	Double^a^	Single	Double^a^	Single	Double^a^	Single	Double^a^
1	27 (14.5)	11 (3.3)	4 (0)	2 (0)	24 (4)	12 (2.4)	32 (6)	15 (2.5)
2	5 (6.9)	2 (2.2)	11 (6.6)	2 (1.7)	5 (6.8)	1 (1.1)	2 (3.6)	0 (0.5)
3	11 (7.3)	1 (1.5)	10 (3.9)	0 (0.5)	6 (4.4)	0 (0.4)	6 (4.5)	0 (0.5)

a D: double sit-to-stands (standing twice in succession).

**Figure 4.**
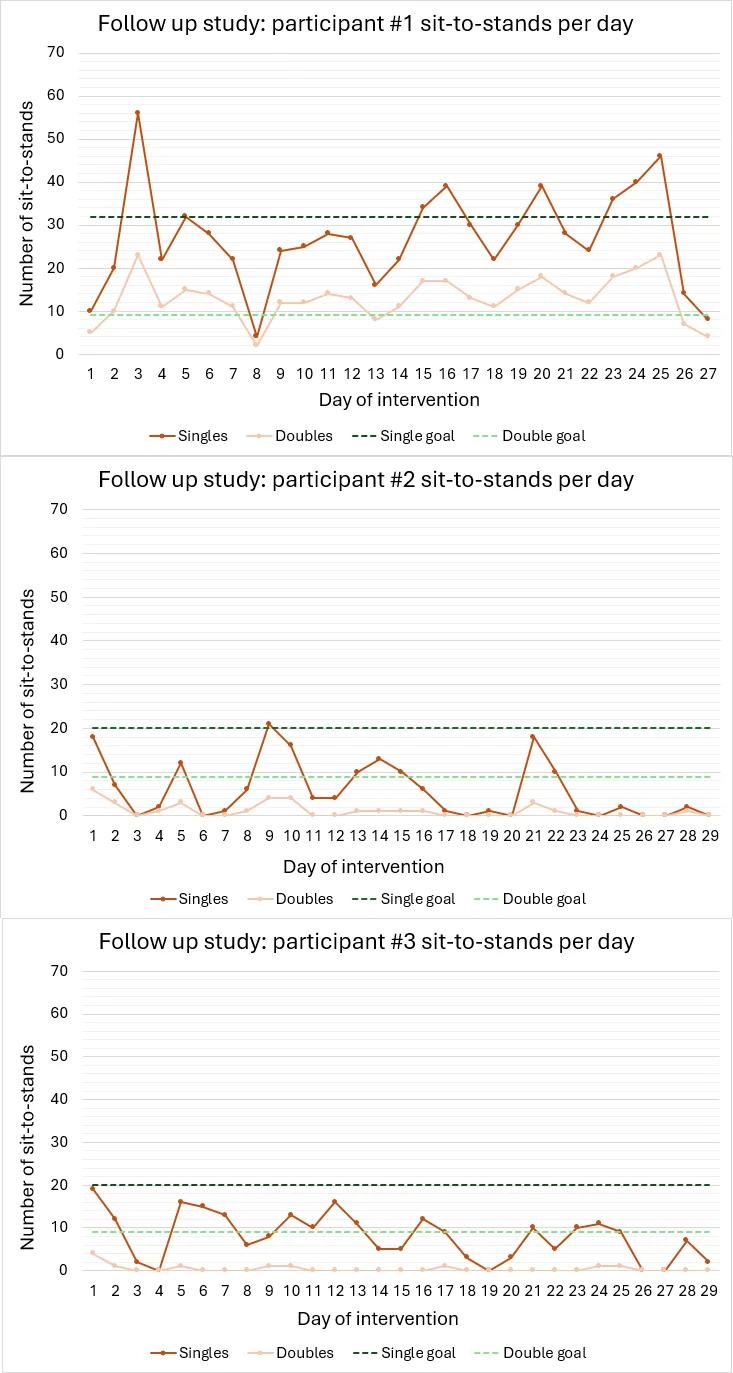
Sit-to-stands per day for the 3 participants in the follow-up study, top to bottom: participant #1, participant #2, and participant #3. Shows single sit-to-stands, double sit-to-stands, and their respective daily goals.

#### Usability Survey Results

All 3 participants rated privacy (P1: 10, P2: 10, and P3: 10) and technical performance (P1: 6, P2: 10, and P3: 10) relatively positively. In addition, P1 rated compatibility (9), dignity and independence (9.33), social engagement (10), emotion (9), and effectiveness (10) all positively. However, P2 and P3 rated similarity (P2: 1.67 and P3: 3.67), dignity and independence (P2: 1.67 and P3: 3), social engagement (P2: 0 and P3: 0), emotion (P2: 0 and P3: 0), and effectiveness (P2: 0 and P3: 1) all relatively negatively. A detailed summary of their usability survey feedback results is included in Table 5.

**Table 5.** Usability results from the 4-week follow-up study.

Participants	Compatibility	Similarity	D and I^a^	Privacy	Social	Emotion	Effective	Technical performance
1	9	3.33	9.33	10	10	9	10	6
2	5	1.67	1.67	10	0	0	0	10
3	7.80	3.67	3	10	0	2.40	1	10

a D and I: dignity and independence.

#### Open Feedback

In his open feedback, P1 found the experience overall very motivating and very enjoyable. He suggested adding a guide to help the user reset the system independently at home. However, P2 expressed that she did not know that she was meant to be more physically active than she normally would and thus felt, in hindsight, that she was using the system incorrectly. And P3 expressed that he continued to act as he normally would during the intervention, standing and sitting when he wanted to.

### Focus Group Results

A total of 25 older adults aged 50 years or older participated in a mix of 6 different focus group or one-on-one feedback sessions held both at the University of Southampton and at a local community center. The 6 sessions were as follows: 3 individual one-on-one sessions held at the university, 2 focus groups held at the university of 3 and 5 participants, respectively, and a focus group held at a local community center of 14 participants. A full participant breakdown is shown in Figure 5.

**Figure 5.**
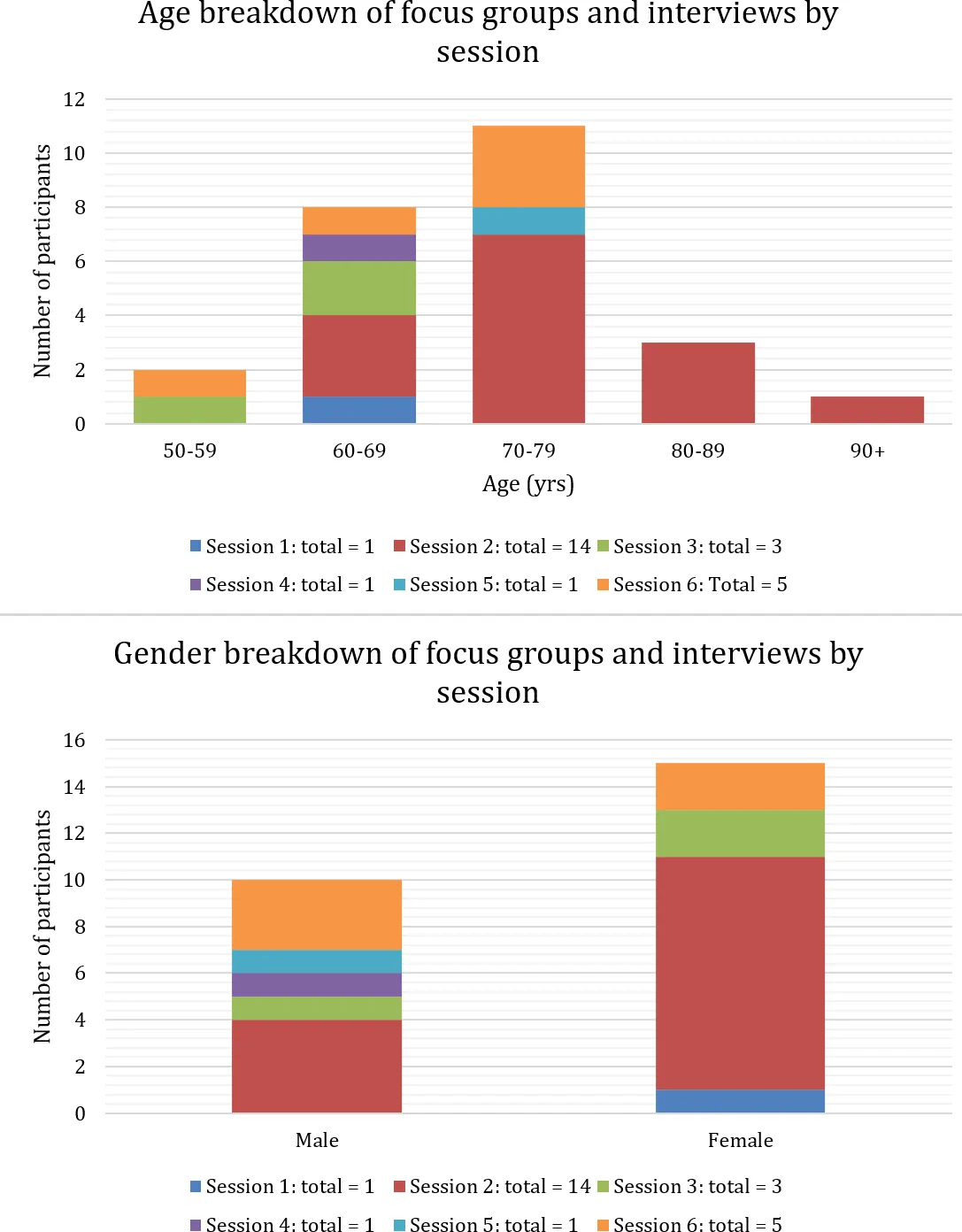
Demographic breakdown (top: age, bottom: gender) of the focus groups and one-on-one interview sessions to gain feedback on potential design ideas and prototypes for the Elder Athlete project.

Overall takeaways from the focus group sessions include a strong preference for the LED light sequence prototype, a preference for smaller, more subtle designs, and a desire for added elements such as sound or music to make the interaction more fun.

A few participants felt that the physicalized prototypes lost some of the functionality present in the screen-based design, such as the daily goals and visual prompting; thus, they preferred the screen-based system to the physicalized prototype. For example, one participant said, “that one, [the screen] tells you, you’ve done one, great, do another. That isn’t so much effort. This one [the physicalized prototype] doesn’t give that same sense of wellbeing.” However, almost all participants preferred the LED sequence design and felt that seeing the current state and future state was sufficient for the vast majority of their anticipated use cases. For example, in reference to the LED sequence, one participant said, “Yeah, I like that. It’s not the figure. It’s the pretty lights,” another added, “personally I like it because it’s not a screen. I’ve already got four screens… Quite useful to have it on the side.” Finally, another said, “A simple thing like that would be [very good]. You don’t need the screen in a sense. The colors will be very good. It’s very bright.”

In general, participants preferred the potential figurines or place cards to be more human-like rather than abstract. One participant reported, “I like more human-like, it would give me more motivation,” another agreed, “[the cardboard figure] was aesthetically pleasing, like an astronaut for example,” and another added, “I think more human-like. I think people will identify with that more, relate to it.”

Finally, participants generally felt that this system could benefit them in terms of helping them be more physically active, though some felt that this would be more beneficial for their parents, friends, or other family members who are less physically active than they are.

In terms of size and location in the home, a concern was brought about by several different participants relating to a feeling of stigma such that they did not want visitors to immediately be able to recognize that this was a medical intervention that they needed to use. For example, one participant, when referencing the cardboard figure sequence said, “That’s quite cute actually… I think that’s way more subtle. It could just be decoration if it was just a light bulb,” they then added referencing the smaller cardboard figures, “[these figures] would work so that visitors would not comment on it, but I would know it was there,” and finally, as general feedback, “Things that don’t look like a hospital or medical aid are better than something that does.” Thus, they preferred smaller designs that could be placed out of the way (eg, not on a coffee table or side table that they use regularly) or on the mantelpiece where only they would know what it is for and could be conceivably part of the home décor.

No concerns of privacy were brought up related to the presented designs, but some privacy concerns were expressed when discussing the potential of a social component that allows users to share activity with friends and family and at the notion that the data might ever be shared with third parties (eg, a distrust of devices such as Amazon’s Alexa). Finally, other design ideas were expressed such as incorporating the intervention into a TV app, using fitness trackers or mobile apps for more detailed information, and designing a music-based system with an interaction functionality similar to “musical chairs.”

The full transcripts of the 6 focus group and one-on-one sessions are included in the Multimedia Appendices 1–3.

## Discussion

### Principal Findings

The discussion will cover the main findings from the case study, its implications on the potential use of the framework for supporting the development of older adult fitness technologies, limitations, and future work.

### Findings From the Pilot Study and Key Insights From the Framework Design Workshops

Prior to using the framework, the research followed a standard design process based on the principles of participatory design [65]. This included conducting outreach surveys with older adults in our community to better understand how they interact with both technology and exercise on a daily basis, one-on-one in-laboratory usability testing to work through initial prototypes to get initial usability and usefulness feedback, and co-design with older adult partners. As evidenced from the pilot study results when compared to factors outlined in the framework, the Elder Smart Chair showed alignment with compatibility, privacy, dignity, and independence, but not with the rest, particularly emotion. Participants scored the Elder Smart Chair an average of 9.33 (SD 1.15) out of 10 on privacy, 6.53 (SD 0.76) out of 10 on compatibility, and 6.56 (SD 1.02) out of 10 on dignity and independence, in comparison to 5.11 (SD 0.84) out of 10 on similarity, 2.33 (SD 2.52) out of 10 on social engagement, and 3.80 (SD 1.06) out of 10 on emotion. Furthermore, qualitative feedback suggested similar findings. Participants found the system useful to them, but also not very challenging. Looking only at traditional usability and feasibility metrics, albeit with a small sample size, the pilot study would be considered successful. The prototype was able to be successfully deployed at home for 2 weeks with little to no technical problems, and participants regularly met their daily goals (more than 85% of the daily goals met on average). However, in terms of interaction behavior, 2 of 3 participants completed their daily goals all in one go every day, and none of the 3 participants interacted with the extra features on the screen; the aforementioned user feedback suggested a design limitation with our approach.

The framework design workshops offered a chance to explore this gap by asking user feedback not typically included in standard usefulness and usability questionnaires [18] and instead focusing on older adult–specific factors [19]. The key insights from these discussions were twofold.

#### Design Workshop Key Insight #1: Positive Reception of the “Do-It-Twice” Approach

First, participants reacted positively to the “Do-it-Twice” framing and liked that the system was lightweight, easy to use, and minimally invasive in their home. Meaning, we had evidence that our prototype had the potential to positively support older adults and that it was worth exploring further and iterating upon. A nontrivial finding, as at this point in the design process, using the framework could also help illuminate when an idea like this had a low likelihood of success and thus was not worth spending more time and resources on.

#### Pilot Study Key Insight #2: Need for Further Development to Foster Motivation and Engagement

The second key insight is that there was something missing in terms of enjoyment for users, motivating them to engage with the system, and finding the right level of difficulty so that the intervention was both challenging and fulfilling for older adults. In our discussions, we identified 2 possible reasons why this was the case, one was simply that our design was not fun, motivating, and exciting enough in its current state, and 2 our design might be better suited for a different population than the one included in our pilot study, and maybe the catch-all term of older adults was not sufficient in defining that population.

Given these insights, two types of design changes were proposed:

1. Simple, immediate changes that did not alter the overall approach of the system and either addressed specific feedback from participants or feedback from the deployment itself. These included removing the standing mat to minimize space requirements (illuminated from the compatibility discussion), updating the training materials with guided images for operating the system and using the screen (illuminated from the similarity discussion), including an option to hide daily goals and put the system in sleep mode (illuminated from the dignity and independence and privacy discussions), and allowing users to adjust their daily goal whenever they wish (illuminated from the dignity and independence discussion).
2. Changes that required further investigation either because we had several options we wanted to test at once or because it was a change to the mode of interaction for participants. These types of changes included exploring physical figurines and LED-based displays as alternatives to a screen (illuminated from the compatibility, similarity, and emotion discussions), adding gamified elements (illuminated from the emotion discussion), and incorporating an optional social feature (illuminated from the social engagement discussion).

For the first type, a follow-up study was proposed to investigate whether the findings would be the same or if the changes were enough to make a positive difference in how the system was perceived by participants. For the second type, conducting focus groups was chosen to allow for more in-depth feedback and the exploration of several design ideas at once.

### Follow-Up Study Results and Its Implications

Feedback from the follow-up study suggests that, with the updated prototype, there was still a gap in our approach specifically related to motivation and, overall, the emotion factor. This can be seen in the average score of 3.80 (SD 4.66) out of 10 in emotion, the relatively low usage rates, and the qualitative feedback from 2 of the 3 participants. The results also gave credence to the hypothesis that our design was incorrectly mapped to a general older adult population. P1, the only participant younger than the typical 60 to 65 years or older adult age range, but who had the lowest initial mobility score (EQ-5D-5L), had consistent usage throughout the 4 weeks, rated the experience relatively high across all factors, and gave feedback that the intervention was extremely enjoyable, motivating, and useful. The results suggest an opportunity for a design decision point, either focus more on sedentary, low-mobility adults (like P1 from the follow-up study) or broaden the interaction to support generalized use by older adults. This form of self-assessment is something that should be iterative and worth exploring with each new stage of the design process. As researchers get more feedback through user studies, pilot studies, and co-design efforts, a part of that process should also be to more specifically hone in on the most suitable target population and make design decisions that align with that choice. While takeaways from our follow-up study are limited by the small sample size, the results have illuminated where our system design refinement should be focused in terms of engagement. To this end, we explored this area explicitly both in terms of design options and in terms of approach to another round of co-design with focus groups, before a next field study.

### Focus Group Results and Its Implications

As shown in the focus group results section, results from the focus group sessions suggested support for the overarching approach of building strength by building practices incidentally through doing it twice by older adults. Participants generally felt that they could see interventions with this approach being useful for them or at least that they knew someone who would find this approach beneficial. Feedback also suggested that the larger design changes identified during the framework design workshops were associated with positive feedback from potential users, specifically the transition from a screen-based design to a physicalized approach. For comparison, participants found the current prototype, the screen-based prototype from the follow-up study, to be less motivating and enjoyable in comparison to the LED sequence-based design. Participants noted its simplicity, its discreteness, and the fun audio and colors. As one participant put it, they felt that they could leave the system displayed in their home, and they would not have to explain it when people came to visit. As in, they liked that only they would know the purpose of the system. This assertion was met with agreement by the rest of the participants in the 5-person focus group and a similar sentiment was shared in several of the other focus groups and one-on-one sessions. Participants were also open to adding social elements such as sharing sit-stand counts with family and friends. However, a few participants expressed concern over privacy related to social features. Overall, there was a belief that the overall approach could be beneficial and a clear preference for the LED sequence-based design.

One takeaway from the focus groups and one-on-one interviews as it pertains to the framework is that, in comparison to our initial usability sessions prior to consulting with the framework, orienting the discussions around the older adult–specific factors helped to stimulate more nuanced and deeper feedback. For example, related to our emotion concern, it was not solely about adjusting the difficulty level, but instead about finding the right population to match, finding ways to bring joy into participating, and finding ways to inspire confidence that participating in this intervention would lead to positive change. Feedback related to the other factors was also nuanced. Most participants were okay with sharing their progress with their family and friends but were wary of sharing any data with third-party organizations and only wanted to share data directly tied to their own health concerns. In addition, as it relates to dignity and independence, a common sentiment was that they did not want the intervention to feel and look like a medical device. If it was something that needed to be displayed in their living room, it should be something that could both blend in for visitors and be an ever-present reminder for themselves.

Without orienting the discussion around the factors in the framework, those nuances may have been missed. However, it is difficult to know exactly which of the feedback would have been found if we had followed other standard participatory design methodologies. However, use of the framework did give researchers a shortlist for some of the most important factors that older adults care about in terms of fitness technologies. Reviews in this space have shown generally that it is not always the same set of the 6 factors: compatibility, similarity, dignity, and independence, etc that the technology interventions miss in their design considerations; however, they generally do miss at least 1 or 2 of them, which could be detrimental to older adults’ willingness to adopt those technology interventions in their daily lives [19]. Further research is necessary to compare use of the framework versus other standard participatory design methodologies without the use of the framework.

### Broader Implications of Using the Framework for Older Adult Fitness Technology Design

Shown in the case study is an example of how use of the Older Adult Fitness Technology Design Framework could help researchers to orient their approach and focus on the specific intended interactions and overall objectives as well as identify potential concerns related to older adult preferences. Focusing on the outcomes from the framework discussion sessions, there was a connection between the identified concerns in the framework design workshop sessions and the user feedback in the follow-up study and focus group sessions. For example, compatibility concerns such as space requirements and environmental obtrusiveness reappeared in the open responses from participants in the follow-up study and in the focus groups, in this case positively toward the LED sequence prototype in comparison to the original prototype. In addition, lower scores related to emotion and motivation identified a key missing aspect of our initial intervention design and thus could help researchers quickly identify one of the potential sources of the lower usage rates during the feasibility studies.

Feedback from the pilot study suggested a gap in our initial approach, and until engaging with the framework, we were unable to more accurately hypothesize the reasons why. Feedback from the focus group sessions identified that a larger shift to our approach was necessary, which only emerged from the richer and more open-ended feedback from potential users and may not have emerged from a strictly usability and usefulness model. In our case, addressing the emotional component meant stripping down the functionalities that weren’t being used, exploring an alternative to the screen or user interface paradigm, and instead adding physical, audio, and visual elements that both added enjoyment to the engagement and maintained the minimal invasiveness.

Use of the framework is meant to be both an iterative and supplemental process, not a replacement for existing design processes and principles such as usability testing, participatory design, and pilot studies. Instead, it is aimed at informing researchers of commonly known factors that older adults have expressly identified as important to them. Thus, it intends to both save time and allow for richer evaluations by forgoing the need to rediscover pain points already known in the field and instead focus on more nuanced discussion particular to the individual technology intervention. In addition, the framework itself is meant to be iterative. While the specific use case in the case study was a “smart chair” for encouraging older adults to be more physically active, we suggest the framework has applicability beyond only this use case to older adult fitness technology interventions in general. The factors by which the framework was developed have been shown to be important factors for older adults when considering whether to use a given fitness technology [19] and the framework is designed for broader use. This has not yet been assessed; however, future work is planned to assess the framework in other use cases in the older adult fitness technology intervention space.

### Limitations and Future Work

Limitations of the study include an overall small sample size and the absence of a control group; thus, results from the study reflect only an illustration of how the framework could be used in practice and should not be seen as validation of the effectiveness of the framework in the broader context of older adult fitness technologies.

Future work is needed to further develop the framework based on the findings from this study and use it in future older adult fitness technology research projects. We plan to use the framework to further develop the Elder Athlete Smart Chair and conduct a larger scale field study with the system. We also plan to develop the framework into an online tool that we will offer to anyone in the research community interested in testing it out and offering their feedback on its use for their older adult health and fitness technology research. We would like this tool to be as useful to the community as possible, so we welcome any feedback on ways to develop it further.

### Conclusions

Presented in this manuscript is the Older Adult Fitness Technology Design Framework and a case study of how the framework could be used in practice using a smart chair. The aim of the framework is to supplement participatory design practice by synthesizing what we already know in the field about older adults related to technology and health and fitness, and is thus intended to allow researchers to identify potential areas for improvement more easily through self-assessment and use their time and resources on deeper and more nuanced evaluation that is specific to their own design application. Thus, synthesized in the framework are known factors that older adults place importance on related to fitness technologies and presented in this manuscript is an example of how the framework could be used in an iterative design process. The framework’s broad use for supporting older adult fitness technology design needs to be explored further; however, at a minimum, the manuscript and the framework itself aim to highlight factors such as compatibility, dignity and independence, and privacy that are of importance to older adults, but are not always explicitly considered in the existing design processes in the field [19].

We encourage researchers to try it and give us their own feedback, so that we can better support researchers in the older adult fitness technology space. The framework can be found in the Multimedia Appendix 4.

## Supplementary material

10.2196/97010 Multimedia Appendix 1 Consent form for the focus group and interview session (ERGO #: 108805).

10.2196/97010 Multimedia Appendix 2 Consent form for the 4-week follow-up study (ERGO #: 99702.A3).

10.2196/97010 Multimedia Appendix 3 Consent form for the 2-week initial study (ERGO #: 95649.A1).

10.2196/97010 Multimedia Appendix 4 Older Adult Fitness Technology Design Framework that was developed and validated in this research.

## Abbreviations

ADHD: attention deficit hyperactivity disorder
CSCL: computer supported collaborative learning
HCI: human-computer interaction
PASE: Physical Activity Scale for Elderly
TUG: Timed Up and Go
