# Supplementary material for: Design Framework for Older Adult Health and Fitness Technologies: Proof-of-Concept Case Study
Source: JMIR Form Res. 2026 Nov 13;10:e97010. doi: 10.2196/97010 (PMC13766584; doi:10.2196/97010)
Supplement: Supplementary file 1 [file formative-v10-e97010-s001.pdf]

ERGO II Ethics application form – FELS

1. Applicant Details

**1.1 Applicant name**	**Dr Isobel Thompson**
**1.2 Supervisor**	**n/a**
**1.3 Other researchers / collaborators (if applicable):** *Name, address, email*	**Dr Chris Tacca** - c.tacca@soton.ac.uk<br>**Dr Arturo Vazquez Alves** - a.vazquez-galvez@soton.ac.uk<br>**Dr Alexander Ng** - a.w.h.ng@soton.ac.uk<br>**Dr Richard Gomer** - r.c.gomer@soton.ac.uk<br>**Dr Martin Warner** - m.warner@soton.ac.uk<br>**Prof Chris Freeman** - cf@ecs.soton.ac.uk<br>**Prof m.c. Shraefel** - mc@ecs.soton.ac.uk

2. Study Details

**2.1 Title of study**	Elder Athletes: Usability testing of an Interactive Incidental Interaction
**2.2 Type of project** (e.g. undergraduate, Masters, Doctorate, staff)	Staff

**2.3 Briefly describe the rationale for carrying out this project and its specific aims and objectives.**
Physical activity has been associated with increased physical and mental health during ageing. However, the 2022 Health Survey for England found that only 10% of older adults over 75 were meeting the standard guidelines for adequate aerobic and muscle strengthening exercise (Neave., 2021). In individuals aged 50 to 70, there is an estimated decline of roughly 30% in muscle strength (Palla et al., 2021). Resistance training is effective in reducing the effects of aging-induced reductions in functional capacity, with enhancements observed in mobility, muscle strength, quality of life and functional independence (Kashi et al., 2023).<br>The research team have developed an at-home incidental interaction. The system uses a 'do it twice' framework, where the user is encouraged to complete sit-to-stand movements, as they usually would when standing or sitting from their couch, twice. This aims to increase their levels of resistance training and physical activity. The current system includes sensors on the seat of the chair, the floor and the two chair arms. The system uses these sensors to identify when the participant stands, when they sit and also when they use their hands on the armrests when completing these motions. A tablet displays the daily number of single and double stands and monitors the user's progress over time to provide continuing motivation for improvement.<br>This round of useability testing follows two previous studies linked to this project. First we completed initial usability (ERGO # 95649.A1), followed by a round of feasibility testing where the systems were deployed in the home (ERGO # 99702.A3). We have now developed an additional system component. This component is designed to be a physical reminder of the sit-to-stand motions which we are looking for individuals to replicate. This takes the form of a figurine, where we have multiple potential designs to work with going forward (differing shapes, sizes and motion). This round of usability will allow us to interact with the target audience of the intervention and gather their thoughts and

opinions of the physical reminder system component in its current state before further development takes place.
The study aims to assess and understand the usability of an at-home incidental interaction system

2.4 Provide a brief outline of the basic study design. Outline what approach is being used and why.

This study is a usability test to assess whether the newly developed system component is viable for use in the home. The study will use exploratory techniques and follow-up questions to gather participants views on the current system component with a view to use their input to suitably develop this further.

2.5 What are the key research question(s)? Specify hypotheses if applicable.

Which features within the system component affect its useability?
Are there any potential barriers that participants can foresee to the implementation of this system component into their home?
How could the current system component be improved, from the user's perspective?

3. Sample and setting

3.1 Who are the proposed participants and where are they from (e.g. fellow students, club members)? List inclusion / exclusion criteria if applicable.

The participants will be older adults (aged over 50 years), consisting of males and females. We will recruit up to thirty individuals for this usability testing study. The following inclusion and exclusion criteria will be applied. The criteria will be outlined in both the participant information sheet and within the consent form, and participants will be asked to confirm that the exclusion criteria do not apply before participation. We will recruit those over 50 due to the time limitations of the study, and our previous difficulties in recruiting those over the 65 year age bracket.

Inclusion	Exclusion
Male and female adults	Adults without sufficient mental capacity to provide informed consent
Over 50 years of age	Adults without sufficient physical capacity to comfortably complete repeated standing and sitting exercises
English speaking	Adults with a speech impediment

3.2. How will the participants be identified and approached? Provide an indication of your sample size. If participants are under the responsibility of others (e.g., parents/carers, teachers) state if you have permission or how you will obtain permission from the third party).

This study will recruit up to thirty participants from social settings and care homes. Recruitment will involve a combination of poster advertisement, and through third parties. Firstly, with the organisation's permission, recruitment posters will be placed in social/community hubs (https://www.southampton.gov.uk/people-places/community-places-rights/) around the surrounding areas. We will ask previous contacts at Abri (housing schemes) and Living Well Hubs Southampton (day care centres) to share this poster at their facilities. All gatekeeper approvals will be uploaded as amendments when granted. The poster will outline the project and inclusion/exclusion criteria to help potential participants decide whether they would like to participate. The poster will include the contact details of the lead researcher so that any potential participants are able to contact the research team and express their interest. Once the potential participant has reached out to the research team, they will be sent a participant information sheet with further information to help them decide if they still wish to participate in the study.<br>Care homes in the surrounding area will also be approached directly. The study will be introduced to the care home, and they will indicate whether they believe there are any potential participants at the home that they would be willing to provide access to. If the care home agrees, they will provide a gatekeeper letter to the research team. They will then be asked to share the study information (PIS) with potential participants, to help them decide if they I wish to participate in the study.<br>A gatekeeper letter in attached to this application for external parties to provide consent to recruit participants. Once gatekeeper approval has been granted, the completed gatekeeper letter will be uploaded in an amendment.<br>During previous studies linked to this project, we have recruited individuals fitting our inclusion criteria who have given permission to be recontacted for future opportunities. We will reach out to these individuals with an invitation to participate in this round of usability testing.<br>Finally, we will also use the VOICE platform to advertise our research study (https://voice-global.org/for-researchers), for which the University of Southampton are a registered user. The advert (attached recruitment documentation) will be live on the VOICE platform for the study duration. The information in the advert directly reflects the information provided in the participant information sheet, and the PIS will also be directly attached to the advert for potential participant's information. Participant's will be able to apply to take part in the study, with the lead researcher receiving a notification from the platform.

**3.3 Describe the relationship between researcher and sample. Describe any relationship e.g., teacher, friend, boss, clinician, etc.**
The individuals proposed for participation are not familiar to the researchers, and there is no pre-existing relationship. Those who have consented to be recontacted may have interacted with members of the research team previously. It will be made clear in the invitation to them that there is no pressure to participate, and that if they are not interested then they should ignore and discard the email.

**3.4 How will you obtain the consent of participants? (***please upload a copy of the consent form if obtaining written consent***) NB A separate consent form is not needed for online surveys where consent can be indicated by ticking/checking a consent box (normally at the end of the PIS). Other online study designs may still require a consent form or alternative procedure (for example, recorded verbal consent for online interviews).**

Once the participant has read the participant information sheet and agreed to take part in the study, a suitable and convenient time will be agreed for the data collection session. When the participant arrives, the lead researcher will make sure that the participant has read the participant information sheet, and will answer any further questions they may have. They will then be asked to sign an informed consent form before the sessions begins.<br>It will be made clear to the participant that they are able to withdraw at any time without giving a reason, by emailing the lead researcher. However, if they withdraw from the study, we will keep any information collected up until the point of withdrawal for the purposes of achieving the objectives of the study only.

**3.5 Is there any reason to believe participants may not be able to give full informed consent? If yes, what steps do you propose to take to safeguard their interests?**
No, there is no reason to believe that anyone without the ability to give informed consent will be recruited, since this is within the exclusion criteria for the study.

4. Research procedures, interventions and measurements

**4.1 Give a brief account of the procedure as experienced by the participant. Make it clear who does what, how many times and in what order. Make clear the role of all assistants and collaborators. Make clear the total demands made on participants, including time and travel.** *Upload copies of questionnaires and interview schedules to ERGO.*
A suitable and convenient time for the data collection session will be confirmed with the participant via email, once they have read the participant information sheet and agreed to take part.<br>The participants will either visit the university of Southampton (Health Sciences, Building 67), or in the case of recruiting through a local organisation or care home, the research team will set up the focus group there. All participants will have the opportunity to ask any further questions they may have before signing an informed consent form. At least two researchers will be present at the data collection session. One will provide introductions and demonstrations, whilst the second researcher will take notes. The session will also be audio recorded using teams, with the video function turned off.<br>Focus groups will be arranged for 5-6 participants at a time. The data collection session will follow the outlined structure below:<br>1. The lead researcher will provide an overview of the study and the goals of the session<br>2. The lead researcher will provide a short introduction to each figurine and then allow the participants to try these out freely.<br>3. Different versions of the figurine will be available at ‘stations’ around the room. Participants will be able to move around these at their own pace. They will be asked questions while they interact with the various versions. A separate researcher will be located at each station to ask questions and take notes.<br>4. Following this period, the participants will come together as a group and the lead researcher will ask them some questions which will form part of a group discussion<br>5. Participants will be given a follow up questionnaire, which they can complete online or on paper format. The questions in the follow up questionnaire will be those listed in the discussion section of the protocol/focus group schedule. This

is in case we do not cover certain points in the discussion time. The survey will be hosted on MS Forms, will be completely anonymous and optional for all participants.

The questions asked during the session are attached, marked ‘demo’ and ‘discussion’ related to points 3 and 4 above respectively. Two functional prototypes of figurines will be displayed (images below), which will move when sensors on the chair system are activated (replicating the motion of sit to stand). Wooden and cardboard figurines will also be used to demonstrate very basic potentials for designs in more ‘humanoid forms’, to see what participants would prefer in terms of shape, size, motion and form of reminder to sit-to-stand.

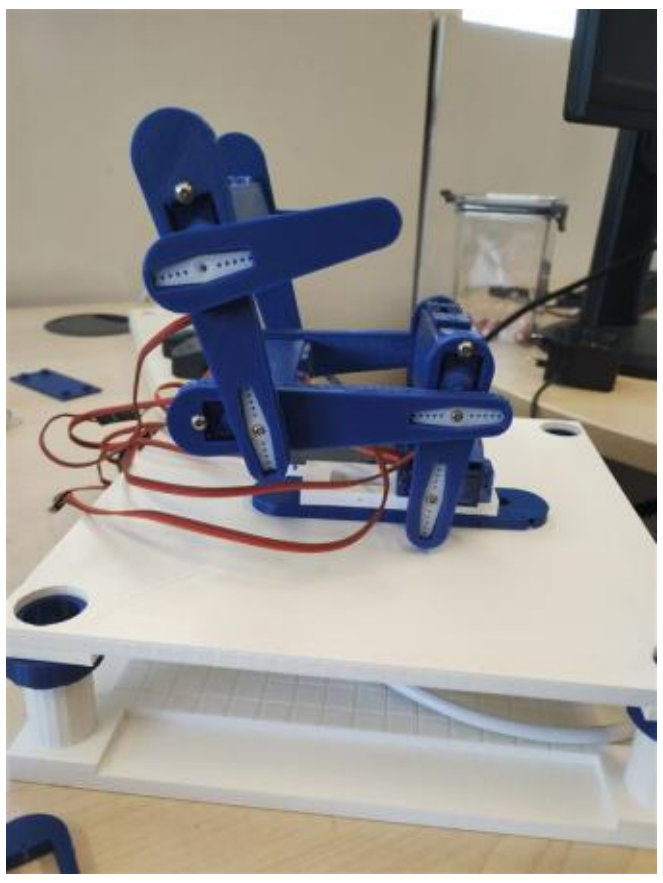

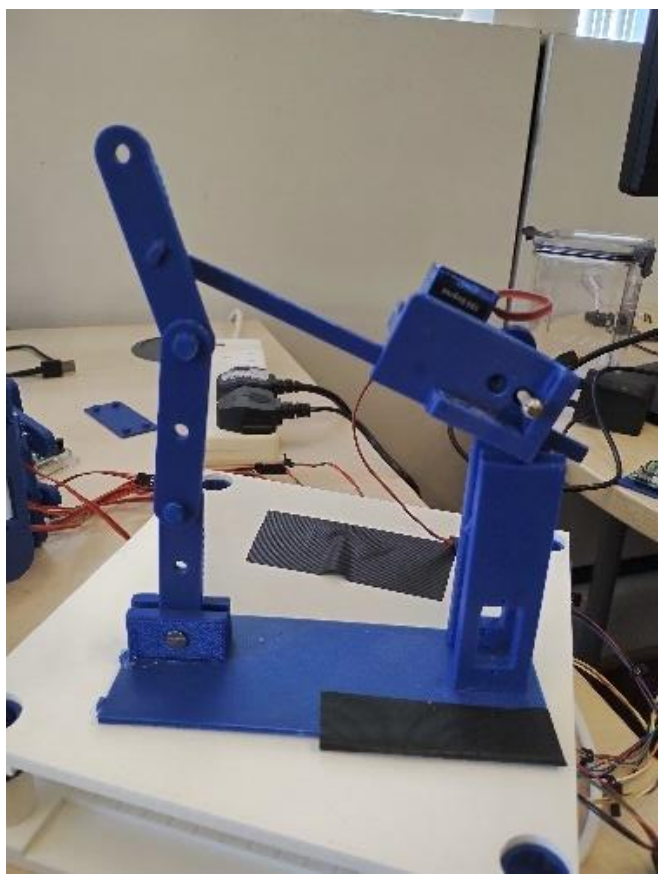

In total, it is expected that this session will last approximately 1 hour. Following the study, a £20 Amazon voucher will be sent to the participants, offered as an incentive for their participation.

**4.2 Will the procedure involve deception of any sort? If yes, what is your justification?**
No, the study will not involve deception of participants

4.3. Detail any possible (psychological or physical) discomfort, inconvenience, or distress that participants may experience, including after the study, and what precautions will be taken to minimise these risks to ensure the well-being, health, and safety of the participants.

Health & Safety policies and resources are available here: https://sotonac.sharepoint.com/teams/HealthSafetyRisk?e=1%3Af902a94a6c14407fbe44e40e5066cb91

If the study involves lone working please state the risks and the procedures put in place to minimise these risks (please refer to the lone working policy). Please seek advice from your local Health and Safety contact if there are significant wellbeing or safety considerations.

Participants may become fatigued during the protocol due to repeated sitting and standing from the chair. They will be asked to confirm that they are physically able to complete the required activity as part of the participant information and consent process. They will also be made aware that they can ask to take a break at any time, and refreshments will be made available to them should they require.

There may be a risk of tripping/falling on cable attachments. The researchers will ensure that the cables are taped down/out of the way. The participants will be made aware of any remaining cables that may present a hazard, although there are not expected to be any that will present a major hazard to participants.

The participants will be reminded that they have the right to withdraw at any time without giving a reason

**4.4 Detail any possible (psychological or physical) discomfort, inconvenience, or distress that YOU as a researcher may experience, including after the study, and what precautions will be taken to minimise these risks and ensure health and safety.**<br>**Health & Safety policies and resources are available here: https://sotonac.sharepoint.com/teams/HealthSafetyRisk?e=1%3Af902a94a6c14407fbe44e40e5066cb91**<br>**If the study involves lone working please state the risks and the procedures put in place to minimise these risks (please refer to the lone working policy). Please seek advice from your local Health and Safety contact if there are significant wellbeing or safety considerations.**
Focus groups will be conducted in person. They will scheduled to occur during work hours in a meeting room at the school of Health Sciences, or within the host organisation setting (providing gatekeeper consent is obtained). The researchers will follow the University's policies, and ensure that colleagues are aware that the focus groups are taking place.

**4.5 Explain how you will care for any participants in 'special groups' e.g., those in a dependent relationship, are vulnerable or are lacking mental capacity), if applicable:**
Not applicable. We are not recruiting individuals within this category. Our exclusion criteria will ensure that only potential participants with sufficient mental and physical capacity will be recruited.

**4.6 Please give details of any payments or incentives being used to recruit participants, if applicable:**
Participants will be offered a £20 Amazon voucher upon completion of the study

5. Access and storage of data

**5.1 How will participant confidentiality be maintained? Confidentiality is defined as non-disclosure of research information except to another authorised person. Confidential information can be shared with those already party to it and may also be disclosed where the person providing the information provides explicit consent. Consider whether it is truly possible to maintain a participant's involvement in the study confidential, e.g. can people observe the participant taking part in the study? How will data be anonymised to ensure participants' confidentiality?**
We cannot guarantee participant confidentiality due to the nature of focus group work. This will be made clear to all participants in the PIS and consent forms. We will ask all participants to agree not to disclose any information discussed during the focus group once they have left.<br>For the purposes of the current study, it is not necessary to link the participant to their data. Anonymous participant data will only be accessible to the named researchers at the University of Southampton through the lead researcher's One Drive.

5.2 How will personal data and study results be stored securely during and after the study. Who will have access to these data?
Audio recordings will be saved to the researcher's OneDrive account. Transcription will be completed by the lead researcher. Upon completion of transcription, all audio files will be destroyed. Electronic data will be stored on the lead researcher's OneDrive account. The data will never be stored on generic/shared computers. Only the other named researchers within the team will be granted access to the electronic files.<br>Paper copies of the signed consent forms will be stored in the lead researchers office within a locked cabinet.<br>Data will be stored for 10 years as per University of Southampton policy.

5.3 How will it be made clear to participants that they may withdraw consent to participate? Please note that anonymous data (e.g. anonymous questionnaires) cannot be withdrawn after they have been submitted. If there is a point up to which data can be withdrawn/destroyed e.g., up to interview data being transcribed please state this here.
It will be made clear both in person and within the Participant Information Sheet that the participant is free to withdraw at any time without providing a reason. They can do so by informing the lead researcher in-person, or by contacting them on i.m.thompson@soton.ac.uk/023 8059 6616. Due to our non data linking approach, it will not be possible to withdraw data once it has been transcribed. If they withdraw from the study after that point, we will keep the information about that we have already obtained for the purposes of achieving the objectives of the study only. This is due to the focus group nature of the study. No identifying details will be collected during the data collection session.

6. Additional Ethical considerations

6.1 Are there any additional ethical considerations or other information you feel may be relevant to this study?
No
