# Supplementary material for: Design Framework for Older Adult Health and Fitness Technologies: Proof-of-Concept Case Study
Source: JMIR Form Res. 2026 Nov 13;10:e97010. doi: 10.2196/97010 (PMC13766584; doi:10.2196/97010)
Supplement: Supplementary file 2 [file formative-v10-e97010-s002.pdf]

University of Southampton

CONSENT FORM

Study Title: Elder Athletes: Assessment of an at-home incidental interaction intervention
Ethics/ERGO number: 99702
Version and date: Version 04, 03/07/2025

Thank you for your interest in this study. It is very important to us to conduct our studies in line with ethics principles, and this Consent Form asks you to confirm if you agree to take part in the above study. Please carefully consider the statements below and add your initials and signature only if you agree to participate in this research and understand what this will mean for you.

Please add your initials to the boxes below if you agree with the statements:

**Mandatory Consent Statements**	**Participant Initials**
I confirm that I read the Participant Information Sheet version 05, dated *03*/07/2025 explaining the study above and I understand what is expected of me.	
I was given the opportunity to consider the information, ask questions about the study, and all my questions have been answered to my satisfaction.	
I agree to take part in this study and understand that data collected during this research project will be used for the purpose of this study.	
I understand that my participation is voluntary and that I am free to withdraw from this study at any time without giving a reason.	

______________________	______________________	______________
**Name of participant**	**Signature**	**Date**
______________________	______________________	______________
**Name of person taking consent**	**Signature**	**Date**

*Once this Consent Form has been signed by all parties, a copy of the signed and dated form should be provided to the study participant. Original signed copy should be stored in the study site file (If applicable).
