# Supplementary material for: Design Framework for Older Adult Health and Fitness Technologies: Proof-of-Concept Case Study
Source: JMIR Form Res. 2026 Nov 13;10:e97010. doi: 10.2196/97010 (PMC13766584; doi:10.2196/97010)
Supplement: Supplementary file 3 [file formative-v10-e97010-s003.pdf]

University of Southampton

CONSENT FORM

Study Title: Elder Athletes: Usability testing of an at-home incidental interaction intervention

Ethics/ERGO number: 95649

Version and date: Version 02, 06/08/2024

Thank you for your interest in this study. It is very important to us to conduct our studies in line with ethics principles, and this Consent Form asks you to confirm if you agree to take part in the above study. Please carefully consider the statements below and add your initials and signature only if you agree to participate in this research and understand what this will mean for you.

Please add your initials to the boxes below if you agree with the statements:

**Mandatory Consent Statements**	**Participant Initials**
I confirm that I read the Participant Information Sheet version 02, dated *06/08/2024* explaining the study above and I understand what is expected of me.	
I was given the opportunity to consider the information, ask questions about the study, and all my questions have been answered to my satisfaction.	
I agree to take part in this study and understand that data collected during this research project will be used for the purpose of this study.	
I understand that my participation is voluntary and that I am free to withdraw from this study at any time without giving a reason.	

Additional Statements - please add your initials in the boxes below you to agree to:

**Additional Consent Statements (if relevant to your study)**	**Participant Initials**
I understand that taking part in this study involves audio recording. I am happy for my interview to be audio recorded and understand that the audio recording will be deleted immediately once transcription is completed.	
I confirm that I am physically fit and able to participate safely in the protocols outlined within the participant information sheet provided to me.	

______________________	______________________	______________________
**Name of participant**	**Signature**	**Date**
______________________	______________________	______________________
**Name of person taking consent**	**Signature**	**Date**

*Once this Consent Form has been signed by all parties, a copy of the signed and dated form should be provided to the study participant. Original signed copy should be stored in the study site file (If applicable).
