# Supplementary material for: Design Framework for Older Adult Health and Fitness Technologies: Proof-of-Concept Case Study
Source: JMIR Form Res. 2026 Nov 13;10:e97010. doi: 10.2196/97010 (PMC13766584; doi:10.2196/97010)
Supplement: Supplementary file 4 [file formative-v10-e97010-s004.pdf]

Elder Fitness Technology Framework

This framework is intended to be a simple step-by-step way for designers to assess how well their fitness/health technologies are aligned with the needs and preferences of elders and plan interventions that are more aligned with those needs.

Part 1: Summarize the interaction of Health Intervention through an Activity Theory Lens

1. Critical Technology
Fill in the technologies that the elder adult will be asked to directly interact with

2. Interaction Type
Fill in what type of experience the intervention will be, ex. game, fitness program

3. Behaviour Lever
Fill in the mechanism of behaviour change: To motivate, inform, and/or make more accessible

4. Goal
Fill in the immediate goal of the health intervention

5. Overall Objective
Fill in the overall objective that the **4. Goal** is trying to address

Fill in the blanks to summarise the health intervention

Using ___________________ (1. Critical Technology) to make a/an ___________________ (2. Interaction Type) to ___________________ (3. Behavioural Lever) elder adults to ___________________ (4. Goal) in order to ___________________ (5. Overall Objective).

Part 2: Assess your current design idea across 8 factors that influence elder adult technology adoption

1. Outcomes

The health intervention should have evidence that it can lead to the desired clinical outcomes as identified in the goal and object in **Part 1: Summary of Interaction**.

What are the established health outcomes for this or similar interventions and what are the validated exercises or programmes for the desired goal?	Does my health intervention have evidence that supports it can lead to the desired health outcomes? List the specific health outcomes here.	If yes, please fill in why here. If no, add the requisite changes to align the intervention with established outcomes. Fill in any assumptions or unknowns for **Part 3: Assessment Plan**.
		**Assumptions/Unknowns:**

2. Similarity with Past Experience

Elder adults are more likely to adopt a technology when they are familiar with the technology they are asked to interact with or the interaction is similar to something they know already.

What are the critical technologies that the user will interact with as identified in Part 1?	Are the intended users familiar with the technology that they will be required to interact with?	If yes, please fill in why here. If no, please adjust the critical technology or add procedures for easier uptake. Fill in any assumptions or unknowns for **Part 3: Assessment Plan**.
		**Assumptions/Unknowns:**

3. Compatibility with Lifestyle

The health intervention should be compatible with the lifestyle of elder adults and should require minimal disruption to daily life patterns.

How will elder adults be asked to interact with the proposed intervention? What will be required of them to participate?	How compatible is the current intervention with elder adults' daily lives? Consider disruptions to routines, setup requirements, and space requirements.	If intervention is suitably compatible, fill in why here. If not, make requisite changes to make intervention more compatible. Fill in any assumptions or unknowns for **Part 3: Assessment Plan**.
		**Assumptions/Unknowns:**

4. Dignity and Independence

Elder adults are more likely to adopt a technology when they feel they are treated with dignity and have more independence. Aspects to consider related to this factor include language used, motivational approach, and agency given to elder adults.

How much agency or control do elder adults have in the health intervention? What are the current design choices made to ensure elder adults are treated with dignity?	Does the health intervention respect elder adults' dignity and promote independence?	If yes, please fill in why here. If no, add the requisite changes to align the intervention. Fill in any assumptions or unknowns for **Part 3: Assessment Plan**.
		**Assumptions/Unknowns:**

5. Privacy Concerns

A major barrier to elder adult technology adoption are privacy concerns. A health intervention should only collect data necessary for it to be effective?

What is data collected in the health intervention? What else is implemented in the design to ensure elder adults' privacy?	Are privacy concerns adequately addressed? Does the intervention only collect data necessary for it to be effective?	If yes, fill in why here. If not, make requisite changes to ensure privacy. Fill in any assumptions or unknowns for **Part 3: Assessment Plan**.
		**Assumptions/Unknowns:**

6. Social Engagement

Some elder adults are more likely to adopt a technology or participate in a health intervention if it gives them an opportunity to be more social and less likely to engage in a health activity if it directly conflicts with their current social engagements. The addition of social aspects to health intervention should be entirely optional to respect elder adults' privacy.

What are the ways in which the current health intervention supports or hinders social engagement?	Does the health intervention provide an option to add social engagement or allow the possibility to be used socially?	If yes, please fill in why here. If no, add the requisite changes to align the intervention. Fill in any assumptions or unknowns for **Part 3: Assessment Plan**.
		**Assumptions/Unknowns:**

7. Emotion

Elder adults are more likely to adopt a technology if it is engaging, motivating, and/or fun to use. Emotion is a key factor for inspiring continued use.

Please fill in the current ways the health intervention is designed to be engaging, motivating, and/or fun to use.	Is the health intervention emotionally stimulating or motivating for users?	If yes, fill in why here. If not, make requisite changes to align the intervention. Fill in any assumptions or unknowns for **Part 3: Assessment Plan**.
		**Assumptions/Unknowns:**

Part 3: Create an assessment plan for the identified goal in Part 1 and address any assumptions identified in Part 2.

List the desired goal identified in **Part 1: Summary of Interaction** and any established clinical outcomes meant to assess that goal. The outcomes can be both behavioural (e.g. retention rate, minutes of physical activity) or clinical (e.g. strength measures, health markers)

List any assumptions identified in **Part 2: Elder Specific Technology Adoption Assessment** and create a plan to explicitly address those assumptions in the assessment.

Propose a short summary of the overall study method here. **Consider:**

1. *What is the intended purpose of the study itself?*
2. *Who are the intended users of this intervention? Be as specific as possible.*
3. *What is the intended duration of use for the technology in practice?*
4. *Could a co-design approach answer some of the assumptions or unknowns in **Part 2: Elder Specific Technology Adoption Assessment**?*
5. *What is the exit strategy for use of the intervention in practice?*
